# Developing a Method to Precisely Locate the Keypoint During Craniotomy Using the Retrosigmoid Keyhole Approach: Surgical Anatomy and Technical Nuances

**DOI:** 10.3389/fsurg.2021.700777

**Published:** 2021-10-08

**Authors:** Zhi-heng Jian, Min-feng Sheng, Jia-yan Li, De-zhu An, Zhi-jian Weng, Gang Chen

**Affiliations:** ^1^Department of Neurosurgery, Zhuhai People's Hospital (Zhuhai Hospital Affiliated With Jinan University, China), Zhuhai, China; ^2^Department of Neurosurgery, Second Affiliated Hospital of Soochow University, Soochow, China

**Keywords:** retrosigmoid approach, transverse sinus, sigmoid sinus, craniotomy, keypoint, digastric groove

## Abstract

**Objective:** To explore the precise location of the keypoint during craniotomy using the retrosigmoid keyhole approach.

**Methods:** This study included 20 dry skulls and 10 wet cadaveric specimens. On the inner surface of dry skulls, the junction between the inferior margin of the transverse sinus (ITS) and the posterior margin of the sigmoid sinus (TSJ) was marked. The keypoint (D) was identified as the TSJ's corresponding point on the external surface of the temporal mastoid process (MP). The distance from the keypoint to the top point of the digastric groove, mastoidale, and asterion were noted (AD, BD, CD, respectively). A method to accurately locate the keypoint was developed based on these relationships. The developed method was used on the wet cadaveric specimens to evaluate its accuracy, safety, rapidity, and minimal invasion.

**Results:** No significant difference was found between the AD, BD, and CD of the left and right sides. The drilling point was oriented on a straight line 12 mm above the top point of digastric groove, perpendicular to the Frankfort horizontal plane (FHP). In the cadaveric specimens, the operative area was clearly exposed. No venous sinus rupture occurred. The average craniotomy time was 28.74 ± 3.89 min.

**Conclusions:** A potentially safe, accurate, and rapid craniotomy procedure was developed with the added advantage of preserving the visibility of the operating field and preventing venous sinus injury.

## Introduction

The suboccipital retrosigmoid approach commonly used in the management of lesions of the cerebellopontine angle including vestibular schwannoma, choleostoma, trigeminal neuralgia, and facial spasm (1–7). It is characterized by a minimal incisions and bone flaps measuring 4 cm and 20 × 25 cm, respectively. It is particularly important to avoid injury of the transverse and sigmoid sinus while maintaining optimal views of the surgical area.

The development of minimal invasive surgery has led to an increased application of the retrosigmoid keyhole approach; this approach emphasizes a proximity between the superior and anterior margins of the bond window with the inferior margin of the transverse sinus (ITS) and the posterior margin of the sigmoid sinus, respectively (6, 8–11). Additionally, the asterion has been considered a keypoint when performing the retrosigmoid approach; however, previous research have associated it with risks and inaccuracy (12). Therefore, the precise location of both the keypoint and bone window is essential during the retrosigmoid keyhole approach but incurs risks when used (13, 14). The keypoint is often located at the corresponding external point of the junction between the ITS and the posterior margin of the sigmoid sinus (TSJ). This study aimed to the develop a new method of orientation to the keypoint and burr hole on the external surface of the mastoid process (MP).

## Materials and Methods

### Specimens and Instruments

This study included 20 dry skulls and 10 wet cadaveric specimens supplied by the Human Anatomy Center of Soochow University and the Second Affiliated Hospital of Soochow University (Suzhou, People's Republic of China). No obvious deformities were observed in the posterior cranial fossae or the petrous and mastoid parts of the temporal and occipital bones.

Zeiss microscopes (OPMI PROergo; Carl Zeiss Meditec AG, Jena, Germany), the SNAKE Microspeed uni-dynamical system (GA B20 DBP; Carl Zeiss Meditec AG), homemade head racks, microscopes, scaleplate (accuracy: 1 mm), and Vernier calipers (accuracy: 0.01 mm) were used.

## Experimental Methods

### The Location of the Keypoint and Measurement of the Lengths in Dry Skulls

**Figure 1:**
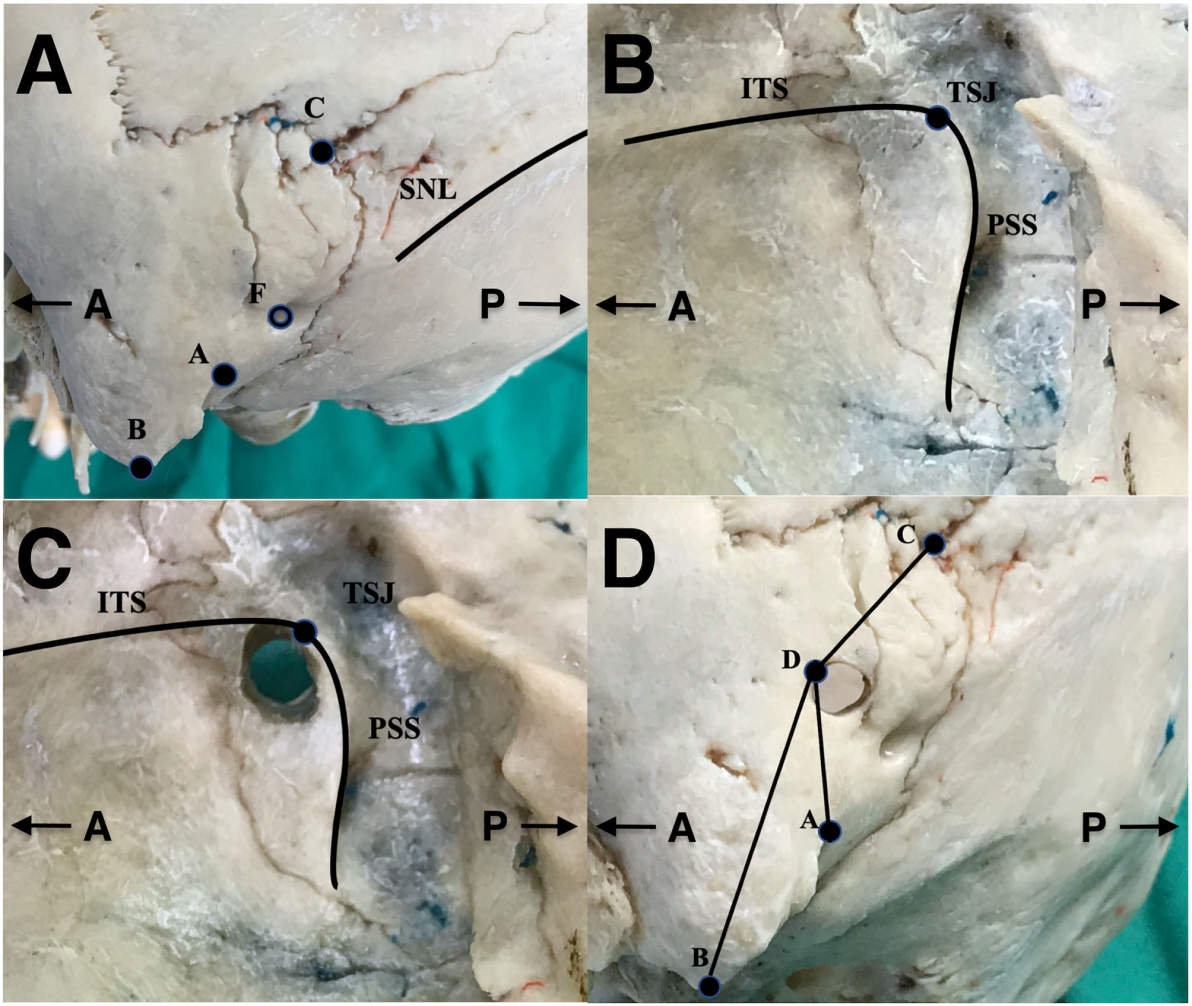
The method used to locate the keypoint in dry skull. **(A)** Identification of extracranial anatomical landmarks on the external surface of the skull, **(B)** identification of the junction between the inferior margin of the transverse sinus and the posterior margin of the sigmoid sinus on the inner surface of the skull (TSJ), **(C)** burr hole from the interior to exterior surface on the basis of TSJ, and **(D)** measuring the length between anatomical landmarks on the external surface of the skull. A, the top point of the digastric groove; B, mastoidale; C, asterion; D, keypoint; F, mastoid foramen; TSJ, the inferior margin of the transverse sinus–posterior margin of the sigmoid sinus junction; SNL, superior nuchal line; ITS, the inferior margin of the transverse sinus; PSS, the posterior margin of the sigmoid sinus.

(1) The following structures were marked on the external surface of the skulls: the top point of the digastric groove (A); mastoidale (B); asterion (C); mastoid foramen (F); and superior nuchal line (SNL) (Figure 1A). (2) On the inner surface of the dry skull, the ITS and the posterior margin of the sigmoid sinus (PSS) were identified; subsequently, the junction between the two structures were marked (TSJ) (Figure 1B). The skull was then drilled vertically, from the interior to the exterior section, based on the TSJ. The diameter of the burr hole was approximately 6 mm (Figure 1C). (3) After the hole was drilled, the corresponding point of the TSJ on the external surface of the skull represented the keypoint (D). (4) The distance of the keypoint from the digastric groove, mastoidale, and asterion were measured (AD, BD, CD, respectively) (Figure 1D). (5) Finally, the method of precisely locating the keypoint in the retrosigmoid keyhole approach was developed according to the finding of the dry skulls.

### Imitation of the Retrosigmoid Keyhole Approach on the Wet Cadaveric Specimens to Verify the Previous Observations in the Dry Skulls

**Figure 2:**
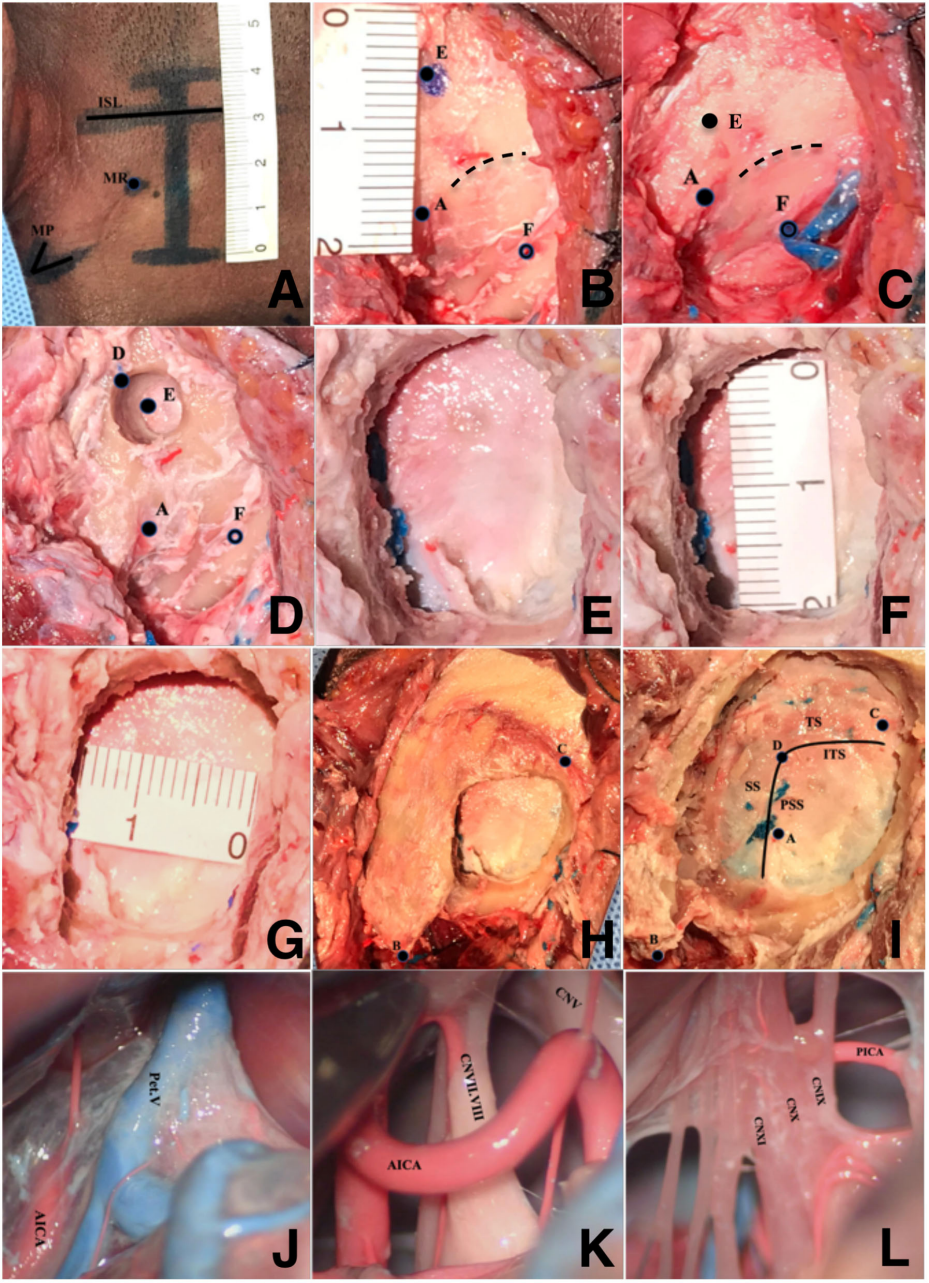
Imitating the new craniotomic method of the retrosigmoid keyhole approach on wet cadaveric specimens. **(A)** Incision of the scalp, **(B)** recognize the bone landmarks, **(C)** confirm the position of the drilling point, **(D)** burr a hole, **(E)** the bone window of the operating area, **(F)** the supero-inferior diameter of the bone window, **(G)** the anteroposterior diameter of the bone window, **(H)** locate the position of the mastoidale and asterion, **(I)** observe the relationship between the anatomical landmarks, and **(J–L**) expose the operating field. A, the top point of the digastric groove; B, mastoidale; C, asterion; D, keypoint; E, drilling point; F, mastoid foramen; MP, mastoid process; MT, mastoid root; ISL, parallel line between the infraorbital margin and the superior margin of the external acoustic meatus; TS, transverse sinus; SS, sigmoid sinus; ITS, the inferior margin of transverse sinus; PSS, the posterior margin of sigmoid sinus; Pet. V, petrosal vein; AICA, anterior inferior cerebellum artery; PICA, posterior inferior cerebellar artery; CNV, trigeminal nerve; CNVII, facial nerve; CNVIII, vestibulocochlear nerve; CNIX, glossopharyngeal nerve; CNX, vagus nerve; CNXI, accessory nerve.

(1) The bony structures, including the external occipital protuberance, MP, and the top point of the digastric groove were marked. A line was drawn to represent the baseline according to the Frankfort horizontal plane (FHP), a horizontal line between the infraorbital and superior margin of the external acoustic meatus (ISL). (2) A straight, vertical incision measuring approximately 4 cm was performed 1.5 cm posterior to the posterior margin of the MP. Approximately one quarter of the incision was made above the FHP. The inferior border of the incision reached the level of the mastoidale (Figure 2A). (3) To recognize the bone structure, the scalp was incised, and the skull was exposed. The top point of the digastric groove, the mastoid foramen, and the SNL were identified on the surface of the skulls (Figure 2B). (4) According to previous observations in the dry skulls, the drilling point was confirmed: 12 mm above the top point of the digastric groove on a straight line perpendicular to the base line (E) (Figure 2C). (5) A burr hole was grounded with the drilling point serving as the central point (diameter: 6 mm). The exact location of the keypoint was 3–4 mm anterosuperior to the drilling point. An oval bone flap (18–20 × 20–25 mm) was made using milling cutters (Figures 2D–G). (6) The dura was incised, and the operative area was exposed to evaluate the revealable satisfaction (Figures 2J–L). (7) The time of craniotomy, the size of bone flap and bone window, and the craniotomy-induced venous sinus injury were evaluated. (8) The scalp incision was enlarged to locate the asterion. The relationship between the asterion and the keypoint was observed (Figure 2H). (9) The bone window was enlarged to orient the transverse and sigmoid sinus, and the top point of the digastric groove (A), the mastoidale (B), the asterion (C), and the keypoint (D). The relationship between the asterion and the keypoint was reconfirmed (Figure 2I).

### Statistical Analysis

The lengths between the related structures were measured using a Vernier caliper and bow compass. The results were expressed as the mean ± standard deviation. Student's *t*-tests were performed using SPSS (Version 21.0; IBM Corporation, Armonk, NY, USA) with significance set at a *P*-value of < 0.05.

## Results

The relationships between the bony structures are shown in Table 1. No significant difference was found between the left and right sides. The relationship of the anatomical structures of the skull specimens demonstrated the following as the accurate method of identifying the drilling point and keypoint: drawing a straight line penetrating the top point of the digastric groove perpendicular to the base line according to the FHP. The center of the burr hole can be exactly oriented 12 mm above the top point of the digastric groove on the vertical line. The keypoint was precisely located 3–4 mm anterosuperior to the drilling point.

**Table 1:** The length between anatomical landmarks in dry skulls (mm).

	**AD**	**BD**	**CD**
Left	16.98 ± 3.58	34.48 ± 3.77	18.49 ± 3.65
Right	15.37 ± 3.25	33.57 ± 3.30	20.89 ± 4.89
t	−0.376	−0.106	0.483
P	0.708	0.916	0.631

*AD, distance between the keypoint and top point of the digastric groove; BD, distance between the keypoint and mastoidale; CD, distance between the keypoint and the asterion*.

In all 10 cadaveric specimens, the craniotomy method identified using the skull specimens was applied and appraised with the following results: (1) Accuracy: Satisfactory exposure of important structures during operation was observed. (2) Safety: Damages to the venous sinuses by the drilling were not observed. (3) Rapidity: the average craniotomy time was 28.74 ± 3.89 min. (4) Minimal invasion: The mean diameter of bone windows ranged from 1.8 to 2.5 and 2.1 to 3.0 cm.

## Discussion

This study aimed to develop of orienting the keyhole and burr hole in the external surface of the MP and found that the most accurate method of identifying the drilling point and keypoint is by drawing a straight line through the digastric groove perpendicular to the FHP.

### The Shortcomings and Risks of Locating the Keypoint on the Base of Asterion

To date, no standardized initial burr hole has been developed due to individual differences of surgeons. Generally, the asterion represented the external landmark and burr hole of the TSJ during the retrosigmoid keyhole approach (15). However, studies refuting the reliability of this landmark for TSJ have been increasing (12, 16–18). Previous studies have found inconsistencies regarding the location of the asterion in relation to TSJ. Particularly, one study found this relationship in only 23.3% of patients; additionally, they found that the asterion corresponds to the ITS in 63.3% of patients (19). Therefore, a risk of venous sinus injury was identified when the asterion was used as the keypoint during craniotomy using the retrosigmoid keyhole approach.

During the retrosigmoid keyhole approach, a straight, vertical, 4 cm incision is made 1.5 cm posterior to the posterior margin of the MP. Limitations to the exposure of the skull is observed leading to non-exposure of the asterion. Scholars have proposed various methods to improve the precise orientation of keypoint. However, the over-exposure of the operating area and the persistence of anatomic variations limit the application of these methods (20–24).

### Locating the Keypoint Using the Top Point of the Digastric Groove

The lack of established standards and maneuverability led to difficulties in clinically applying the novel method. Therefore, we explored a new method to address this problem based on our clinical experience and previous anatomical research. Dry skulls and wet cadaveric specimens were used to standardize this method. The FHP was selected as a base line, and a straight line was drawn through the top point of the digastric groove, perpendicular to the base line. Then, the burr hole was confirmed: the center (drilling point) of the burr hole can be exactly oriented 12 mm above the top point of the digastric groove on the vertical line. Following this, the burr hole was drilled (diameter: 6 mm) on the center point. The keypoint was accurately located at 3–4 mm anterosuperior to the center. Finally, an oval bone window of 15–20 mm (vertical diameter) was made by using a milling cutter. This method could accurately locate the keypoint with good visibility of the operating area and avoid injury to the venous sinuses and the unnecessary removal of bone. Considering the 3–4 mm distance of the TSJ point to the drilling point, the use of a 1-mm carborundum drill was suggested to avoid damages to the venous sinus.

### Locating the Keypoint Assisted by Other Landmarks

The mastoidale is the most prominent landmark used in the retrosigmoid keyhole craniotomy approach. After exposure of the skull's surface, preliminary confirmation of the keypoint was performed through orientation of the top point of the digastric groove. Subsequently, further modifications were performed on the keypoint according to its distance to the mastoidale for precise location of the keypoint. The mastoid emissary vein is a traffic vein linking the skull interior and exterior. The internal opening is at the center of the vertical part of the sigmoid sinus, while the external opening is at the mastoid foramen outside the skull. The mean length of the mastoid emissary vein inside the MP was 11.77 ± 3.19 mm. Anatomic variations were observed in the mastoid foramen. The mean diameter of the mastoid emissary vein foramen was 2.15 ± 0.81 mm occurring at a rate of 61%. The number of mastoid emissary veins foramen ranged from 1 to 4; however, it is consistently located anterior to the occipitomastoid suture (25, 26). The significantly high rate of variability of the mastoid foramen leads to difficulty in precisely locating the keypoint; however, the keypoint is always anterosuperior to the mastoid foramen. Further modifications were made after locating the keypoint according to the digastric groove and mastoidale to precisely locate the keypoint in its anterosuperior relation to the mastoid foramen. The SNL is located between the external occipital protuberance and mastoidale; it is a cambered osseous eminence where the occipital muscles attach to. The lateral part of the SNL is located below the level of the transverse sinus and the length between the SNL and transverse sinus ranges from 1.5 to 14 mm (27). Due to the aforementioned anatomic relationship, the burr hole can be located 12 mm vertically above the top point of the digastric groove. Further modifications were made to the keypoint according to its distance from the mastoid to precisely locate the keypoint. Subsequently, further modifications were made according to the relationship between the venous sinus and mastoid foramen/SNL. These modifications provide the accurate location of the keypoint: anterosuperior to the mastoid foramen, above the lateral portion of the SNL. Due to the limitation of the sample size, our study were not comment on the variation of the digastic groove and potential effect of them. However, the result are satisfactory and we will concern on our further study.

## Conclusion

In our research of the modified retrosigmoid keyhole craniotomy, anatomical structures in the operative scope were used to quantify the length between the top point of the digastric groove, the mastoidale, and the keypoint. Based on the relationship between these anatomical structures of skull specimens, the following method was identified to accurately locate the burr hole: a straight line drawn through the top point of the digastric groove, perpendicular to the base line according to the FHP. The exact center of the burr hole can be vertically oriented 12 mm above the top point of the digastric groove. The keypoint was accurately located 3–4 mm anterosuperior to the center. Simultaneously, further modifications were made to the keypoint to identify its exact location anterosuperior to the mastoid foramen and above the lateral part of the SNL. This method can help in the accurate localization of the TSJ to ensure a safe, accurate, and rapid craniotomy with an adequate view of the operating area and avoiding injury to the venous sinus. Further studies using the new method should focus on its application in neurosurgery to develop the method leading to ease of performance.

## Data Availability Statement

The original contributions presented in the study are included in the article/Supplementary Material, further inquiries can be directed to the corresponding author/s.

## Ethics Statement

This study is an anatomical study and did not involve any human participants. The studies were reviewed and approved by the Ethics Committee of Zhuhai People's Hospital.

## Author Contributions

GC contributed to the study concept and design. M-fS, J-yL, and D-zA contributed to the acquisition of data. Z-jW contributed to the analysis and interpretation of data. Z-hJ contributed to the drafting of the manuscript. All authors read and approved the final manuscript.

## Funding

This project was funded by Zhuhai People's Hospital scientific research initiation project no. 2021KYQD-02 to GC.

## Conflict of Interest

The authors declare that the research was conducted in the absence of any commercial or financial relationships that could be construed as a potential conflict of interest.

## Publisher's Note

All claims expressed in this article are solely those of the authors and do not necessarily represent those of their affiliated organizations, or those of the publisher, the editors and the reviewers. Any product that may be evaluated in this article, or claim that may be made by its manufacturer, is not guaranteed or endorsed by the publisher.
